# Exploring Microbial-Based Green Nanobiotechnology for Wastewater Remediation: A Sustainable Strategy

**DOI:** 10.3390/nano12234187

**Published:** 2022-11-25

**Authors:** Sumira Malik, Archna Dhasmana, Subham Preetam, Yogendra Kumar Mishra, Vishal Chaudhary, Sweta Parmita Bera, Anuj Ranjan, Jutishna Bora, Ajeet Kaushik, Tatiana Minkina, Hanuman Singh Jatav, Rupesh Kumar Singh, Vishnu D. Rajput

**Affiliations:** 1 Amity Institute of Biotechnology, Amity University Jharkhand, Ranchi 834001, Jharkhand, India; 2 Himalayan School of Biosciences, Swami Rama Himalayan University, Jolly Grant, Dehradun 248140, Uttarakhand, India; 3 Institute of Advanced Materials, IAAM, Gammalkilsvägen 18, 59053 Ulrika, Sweden; 4 Mads Clausen Institute, NanoSYD, University of Southern Denmark, Alison 2, 6400 Sønderborg, Denmark; 5 Research Cell & Department of Physics, Bhagini Nivedita College, University of Delhi, New Delhi 110043, India; 6 School of Science, PP Savani University, Surat 394125, Gujrat, India; 7 Academy of Biology and Biotechnology, Southern Federal University, 344090 Rostov-on-Don, Russia; 8 NanoBioTech Laboratory, Health System Engineering, Department of Environmental Engineering, Florida Polytechnic University, Lakeland, FL 33805, USA; 9 Department of Soil Science and Agricultural Chemistry, S.K.N. Agriculture University, Jaipur 303329, Rajasthan, India; 10 Centre of Molecular and Environmental Biology, Department of Biology, Campus of Gualtar, University of Minho, 4710-057 Braga, Portugal; 11 InnovPlantProtect Collaborative Laboratory, Department of Protection of Specific Crops, Estrada de Gil Vaz, Apartado 72, 7350-999 Elvas, Portugal; 12 School of Engineering, University of Petroleum and Energy Studies (UPES), Dehradun 248007, Uttarakhand, India

**Keywords:** bioaugmentation, biosorption, bioremediation, bio-nanotechnology, adsorptive performance, catalytic performance

## Abstract

Water scarcity due to contamination of water resources with different inorganic and organic contaminants is one of the foremost global concerns. It is due to rapid industrialization, fast urbanization, and the low efficiency of traditional wastewater treatment strategies. Conventional water treatment strategies, including chemical precipitation, membrane filtration, coagulation, ion exchange, solvent extraction, adsorption, and photolysis, are based on adopting various nanomaterials (NMs) with a high surface area, including carbon NMs, polymers, metals-based, and metal oxides. However, significant bottlenecks are toxicity, cost, secondary contamination, size and space constraints, energy efficiency, prolonged time consumption, output efficiency, and scalability. On the contrary, green NMs fabricated using microorganisms emerge as cost-effective, eco-friendly, sustainable, safe, and efficient substitutes for these traditional strategies. This review summarizes the state-of-the-art microbial-assisted green NMs and strategies including microbial cells, magnetotactic bacteria (MTB), bio-augmentation and integrated bioreactors for removing an extensive range of water contaminants addressing the challenges associated with traditional strategies. Furthermore, a comparative analysis of the efficacies of microbe-assisted green NM-based water remediation strategy with the traditional practices in light of crucial factors like reusability, regeneration, removal efficiency, and adsorption capacity has been presented. The associated challenges, their alternate solutions, and the cutting-edge prospects of microbial-assisted green nanobiotechnology with the integration of advanced tools including internet-of-nano-things, cloud computing, and artificial intelligence have been discussed. This review opens a new window to assist future research dedicated to sustainable and green nanobiotechnology-based strategies for environmental remediation applications.

## 1. Introduction

In recent decades, the advancement in the field of nanoscience and nanotechnology has led to improved treatment procedures for wastewater. The various nanotechnological-based pathways have been proven to be more effective than the traditional treatment techniques [1]. Nanoparticles (NPs) are smaller in size, have higher chemical characteristics, and are known to have a broad surface area-to-volume ratio. Traditionally, the NPs were synthesized using physical and chemical methods. These methods involved the application of hazardous materials, sophisticated machinery, and high-end equipment. This made the process of synthesis expensive, and difficult, and had a detrimental effect on the environment [2]. SSF slow filters accompanying biofilms with specific bacterial inoculum in the removal of biologically originated pathogens from streams, rivers, or lakes water bodies for beneficial purposes in agriculture and domestic use [3,4]. To overcome these downsides a significant shift towards a more feasible and environmentally friendly, microbial-based green NT is taking better shape. Green synthesis, as part of bio-inspired procedures, offers consistent and sustainable methods for the biosynthesis of NPs. This field is developing enormously and new methods in this field are constantly being designed to improve the existing properties of NPs [5].

Through a straightforward and eco-friendly process, a porous hierarchical based membrane was produced using a superior nanotechnological technique, demonstrating excellent flux and separation properties for oil/water emulsions, and dye removal was accomplished through photodegradation and adsorption. These nanomembranes exhibits anti-oil/dye/microorganism-fouling potential and these membranes efficaciously separates complex wastewater [6,7]. A hierarchical, fluorine-lose, and robust superhydrophobic membrane was constructed using electrospinning, rapid deposition, and soaking. The membrane had good self-cleaning, superhydrophobic, and oil/water separation capabilities [8]. Electro spun fiber membranes have overcome the drawbacks of traditional wastewater treatment methods, such as high energy consumption, low efficiency, and difficulty recycling. By combining pre- or post-functionalization methods with electrospinning, nanofibers can be produced with an ion-exchange functionality [9]. Microorganisms are imperative nano-factories capable of gathering and detoxifying heavy metals (HMs) [6]. They consist of a variety of reductase enzymes that help in the reduction of metal salts to NPs. The synthesis of green nanomaterials (NMs) from microorganisms has paved a path toward the eco-friendly remediation of pollutants. For example, the iron NPs extracted from *Ageratum conyzoides* are used in the remediation of wastewater due to their redox potential. They also react with water, provide magnetic susceptibility, and are non-toxic [10].

Similarly, membrane-associated NMs are also an effective method for effluent removal. They benefit by improving the membrane permeability, mechanical and temperature strength, resistance, and various functioning for the degradation of pollutants [11]. Nano-catalysts are well known for their role in the enhancement of degradation reactions. The photocatalytic nanofiber-based membranes are antimicrobial, cost effective and have application in micropollutants degradation with removal of harmful organism that consequently results in reduction of fouling of membrane [12]. Au/TiO_2_ photocatalyst treats Lomefloxacinin antibiotic contaminated wastewater [13].

Apart from membranes and nano-catalysts, metal-organic frameworks (MOFs) are active in the removal of HMs from wastewater. The coordination of organic ligands with precursors of metal ions leads to the synthesis of these MOFs [14]. The presence of functional groups with metal acts as a hindrance to the organic ligand. This reduces the steric hindrance and helps in the synthesis of extracellular green metal NPs. The NMs synthesized by this method are of more importance because they replace the need for expensive complex downstream processing steps [15]. It makes the recovery of extracellular NPs much easier. Biogenic NPs and nanomachinery designed through the microbial cells, either directly or their byproducts, e.g., cell proteins, and metabolites, are used to synthesize NPs for the bioremediation of the toxic pollutants from wastewater [16]. The green chemistry methods for the of NPs synthesis involve the use of live microbial cells be an innovative and effective tool in bio-nanotechnology.

The biogenesis of the NPs is eco-friendly, sustainable and cost-effective results highly efficient and productive products [17]. Such NPs possess significant sorption and catalytic efficiency and reduce the procedure cost for large-scale treatment of water bodies. In recent decades, different methodologies for the bioaugmentation, immobilization, and entrapment of microbial cells NPs showed highly effective and increased reaction rate bioremediation processes, e.g., such as metal oxides NPs, biopolymer-coated metal NPs, nanocomposites [18,19].

**Figure 1:**
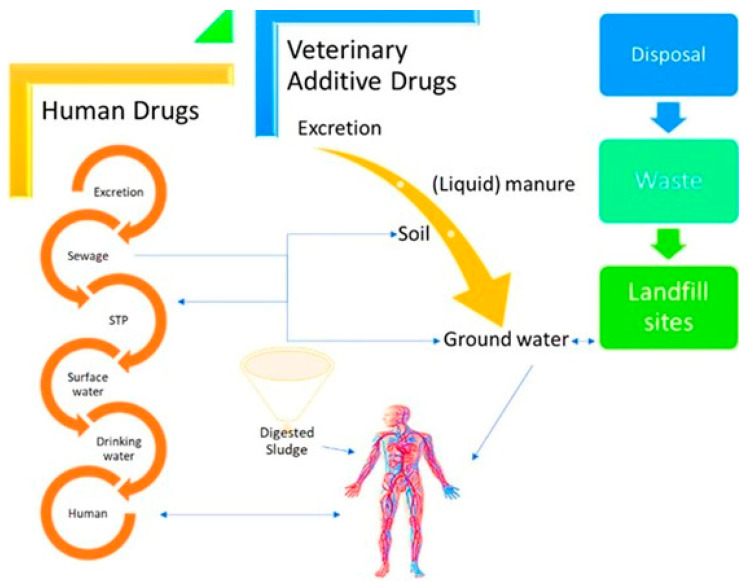
The different sources of contaminants in the wastewater system [24].

Wastewater is contaminated from different sources as explained in Figure 1. The different sources of contaminants in the wastewater system In natural remediation microbial cell and their consortium play an essential role to naturally metabolizing these toxic pollutants into the less toxic form, e.g., the saprophytic nature of fungi [20]. Thus, researchers have focused on microbes having bioremediation and bioconversion properties. Furthermore, lots of work such as bioreactor systems with the integration of NT, NPs entrapped catalytic membrane bioreactors, microbial fuel cells (MFCs), aerobic digesters, and nanofibrous matrix have been done in the past few years to significantly improve the efficiency of remediation of pollutants from the wastewater [21,22,23]. Overall, green NPs are a great alternative to the existing ways of NPs synthesis.

In this review article, the latest advances, and innovations in the production of metal NPs using green synthesis by different groups of microorganisms and the application of these NPs in wastewater treatment sectors are discussed. We have also focused on removing dyes and pharmaceutical pollutants from the wastewater using such green or biogenic NPs. The review article also discusses the advanced integrated methodologies, major challenges, and the future prospects for improving the efficiency of removal of such pollutants from wastewater.

## 2. Nanotechnological Approaches for Wastewater Treatment

Clean and safe water is a fundamental human need for the multi-faceted development of society and a thriving economy. Measures are being taken to reduce this existing situation. Wastewater generated from domestic as well as industrial sources is both equally decremental to the environment [25]. To combat this issue mostly chemicals are employed which can have to the already growing concern over wastewater treatment. The treatment procedure should be effective, environmentally friendly, and conveniently cost-effective. Nanomaterials are known to be successful in the removal of various pollutants from wastewater [26]. This includes HMs organic and inorganic solvents, color as well as biological toxins, and pathogens. In recent years, NT has become a sustainable and eco-friendly approach for the treatment of water and other environmental remediations [2]. Using this system physiochemical and structural modification of the commonly used materials has been done for the efficient adsorption, degradation, and removal of toxic pollutants from the water bodies.

A wide range of engineered NMs and devices have been designed at a molecular and atomic level such as biosorption, dendrimers, nano-filters, and photocatalysis, thereby improving the efficiency of the water treating systems [27]. Besides that, different types of composite and integrated treating systems or filters consist of chemical and biogenic NPs, e.g., metallic, bimetallic NPs, mixed oxides, zeolites and carbon nanotubes/wires (CNT/Ws) are designed to increase the surface to volume ratio, better reactivity and neutralization of the toxicants with significant recovery rate [28]. The use of NT/NPs involves using nanomembranes to soften the water and eradicate biological and chemical contaminants as well as other physical particles and molecules. In this regard, nanobiotechnology has emerged as a subsidiary field focusing on the use of microbes and their active catalytic molecules as bio-nanomachinery for the synthesis and entrapment of NPs for bioremediation.

**Figure 2:**
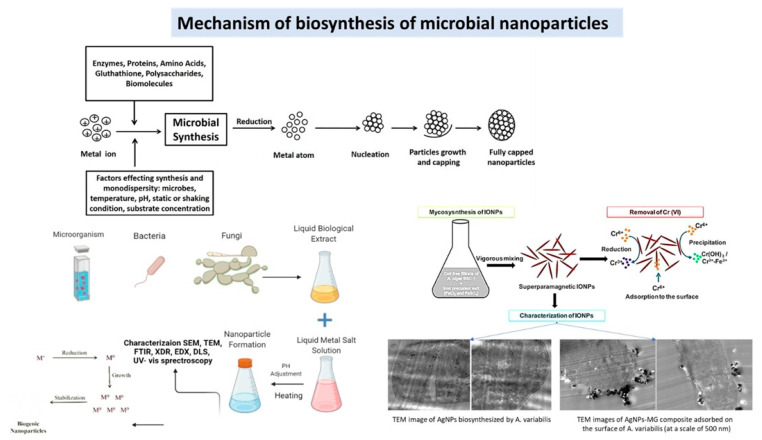
Mechanism of biosynthesis of microbial nanoparticles and their analysis for treatment [39].

Microbes such as bacteria, fungi, algae, and their enzymes are used for the bioremediation of several toxic pollutants in wastewater (Figure 2). Previous studies reported the presence and decontamination of synthetic dyes in the effluents and their combinatorial treatment through physical, chemical and biological approaches with novel technology [29]. The application of microbes is considered a sustainable and renewable resource that is easy to cultivate, and cost-effective technology for large-scale production [30]. The NPs produced by microbes or biogenic NPs effectively degrade and remove HMs, toxic dyes, pharmaceuticals, and other emerging contaminants released by industrial, agricultural, and municipal waste in the water bodies (Table 1). The scope of use NPs in wastewater treatment is just not limited to the removal of chemical and HMs pollutants however, it is also promising for the early detection of the pathogen, toxicants and disinfection by-products (DBPs) by sensing technology [31]. So, it is thought that NT could offer affordable and accessible clean water solutions to the world’s most vulnerable populations.

**Table 1:** Bio-nanoparticles for removal of wastewater pollutants.

Microorganisms	Biosynthesized NPs	Pollutant	Size Range (nm)	Characterization	Removal Efficiency	Mechanism of Pollutant Removal	References
	**Heavy metals**						
Manglicolous fungi	Iron oxide nanoparticles	Cr (VI)	2–16	UV-vis spectroscopy, FTIR spectroscopy, FESEM-EDX, TEM-EDX, XRD analyzer, VSM	>90%	Chemisorption	[32]
*Aspergillus niger* BSC-1	Iron oxide NPs		20–40	UV-vis spectroscopy, ATR-FTIR spectroscopy, Raman spectroscopy, XRD, TEM, FESEM, zeta sizer, VSM	>99%	Adsorption and redox reactions	[33]
*Aspergillus terreus* S1	Magnesium Oxide NPs		8.0–38.0	UV-vis spectroscopy, FTIR spectroscopy, TEM, SEM-EDX, XRD, DLS	97.5%	Precipitation and adsorption	[34]
*Marinobacter* sp. MnI-79	Manganese oxide NPs	Ag^+^	-	XPS, surface analyzer	95%	Electrostatic attraction and redox reactions	[35]
*Streptomyces thermolineatus*	Iron Oxide magnetic NPs	Cu	22	UV-vis spectroscopy, FTIR spectroscopy, SEM, TEM, XRD, thermogravimetric analysis, vibrating sample magnetometer, dynamic light scattering.	85%	Interactions between electrostatic attraction, surface complexation, and coordination	[35]
*Pseudomonas aeruginosa* JP-11	Cadmium Sulphide NPs	Cd(II)	20–40	UV-vis spectroscopy, FTIR spectroscopy, XRD, FESEM, TEM, AAS	88.66%	Adsorption	[26]
*Spirulina plantesis*	Palladium NPs	Pb	10–20	UV-vis spectroscopy, XRD, FTIR, TEM	90%	Adsorption	[36]
*Aspergillus tubingenesis* STSP 25	Iron Oxide NPs	Pb(II)	73.05	UV-vis spectroscopy, Zeta analyser, DLS, FTIR, TEM-EDX, XRD, SQUID-VSM	98%	Adsorption	[32]
		Ni(II)			96.45%		
		Cu(II)			92.19%		
		Zn(II)			93.99%		
	**Dyes**						
*Shewanellaoneidensis*	Magnetite/reduced graphene oxide nanocomposite	Methylene blue	11.0	TEM, XRD, FTIR spectroscopy, XRP spectroscopy, vibrating sample magnetometry	100%	Electrostatic attraction	[37]
*Caldicellulosiruptorsaccharolyticus*	Palladium NPs	Methyl orange and Diatrizoate	10–20	AAS, SEM, TEM-EDS	100%	Reduction	[37]
*Bacillus marisflavi* TEZ7 [38]	Silver NPs	Direct Blue-1, Methyl Red & Reactive Black 5	11.20–39	FTIR Spectroscopy, XRD, TEM, SEM, EDS	54.14–96.92%	Photocatalytic degradation	[38]
*Bacillus paralicheniformis, Bacillus pumilus, Sphingomonaspaucimobilis* [39]	Silver NPs	Malachite green	4–20	XRD, TEM, FTIR spectroscopy	>90%	Adsorption	[39]
*Spirulina plantensisn*			17.9	UV-vis spectroscopy, XRD, TEM, FTIR spectroscopy	88%	Biosorption	
*Anabaena variabilis* [39]			26.4		81%		
*Pseudochrobactrum* sp. C5	Zinc Oxide NPs	Methanol blue and reactive black 5	90–110	FTIR spectroscopy, XRD, FESEM	>90%	Catalytic degradation	[39]
**Pharmaceutical and Hospital wastewater contaminants**							
*Shewanellaoneiedensis* MR-1	Bio-Palladium NPs doped with Au(0)	Diclofenac	-	-	43.8 ±5.5%	Catalytic degradation	[40]
*Pseudonomas putida*	Manganeese Oxide NPs	Estrone and 7α-ethinylestradiol	-	TEM-EDS, HPLC-MSMS	100%	Absorption and oxidation	[41]
*Desulfovibrio vulgaris*	Platinum NPs	17-β estradiol	-	TEM	94%	Catalytic reduction	[39]
		Sulfamethoxazole			85%		
*Escherichia coli*	Biogenic Palladium NPs	Ciprofloxacin	10–30	SEM, EDXA, XPS	87.70%	Reductive degradation	[38]

**Figure 3:**
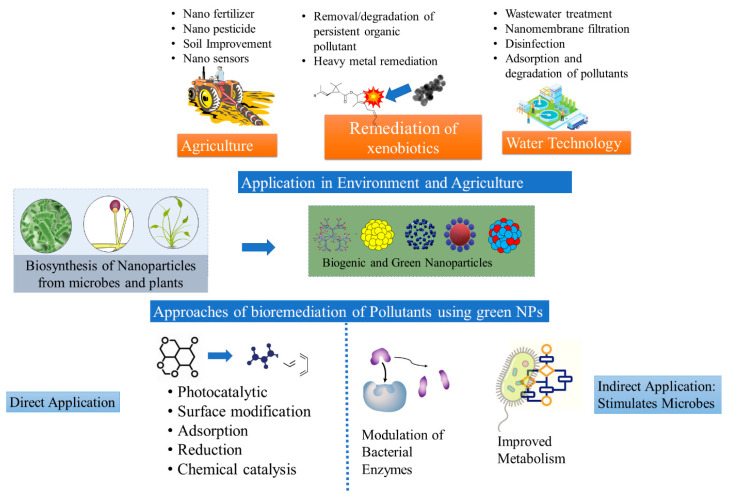
Biogenic Nanoparticles and their application in environment and agriculture (**top**) the bioremediation approach for the removal of toxic pollutants from the environment (**bottom**) [47].

NPs are synthesized using metabolites and cell components of microbial cells, including bacteria, fungi, actinomycetes, and microalgae, by activating or stimulating intracellular or extracellular pathways and used in wastewater treatment [42]. Fungus like *Aspergillus tubingensis*, *Avicennia officinalis, Chlorella vulgaris* are already used in the synthesis of iron NPs. The microbial cell enzymatic machinery reduced the entrapped metal ions and macromolecules accumulated on the cell surface or intracellular regions into NPs [43]. The biogenesis of NPs required additional downstream treatments for the purification and isolation of NPs, e.g., lysis of cells by ultrasonic, irradiation, flocculation, and rectification of NPs (Figure 3). In some cases, the metal resists proteins exhibited the reduction and capping of NPs to overcome the aggregation of NPs and enhance their stability for a prolonged incubation period. Researchers have shown the bioreduction and capping of potassium permanganate (KMnO_4_) into manganese dioxide (MnO_2_) NPs using *Saccharomyces cerevisiae* cellular machinery [44]. Subsequently, Silver NPs (AgNPs) were synthesized from cell-free extracts of different microbial strains, i.e., *Bacillus paralicheniformis*, *Bacillus pumilus and Sphingomonas paucimobilis*. The microbial enzymes reductase and bioactive metabolic peptides assist the stabilization of NPs, subsequently biosynthesized AgNPs display up to 90% efficiency for the removal of dye from wastewater [45]. Likewise, the biosynthesis of chromium NPs (CrNPs) was done by the Cr-resistant *Bacillus subtilis* isolated from the electroplating effluent waste. These CrNPs exhibited antipathogenic activities against Gram-negative and positive bacteria strains, i.e., *Staphylococcus aureus* and *Escherichia coli* [46].

### 2.1. Removal and Recovery of Heavy Metals

Microbes aids the treatment of multiple pollutants through doping of nanoparticles to improvise their action of biological process or bioremediation during the treatment. However, toxicity of nanoparticles is an issue. Therefore, to address the same nontoxic nanomaterials perhaps which increases microbial application in wastewater treatment must be used [48]. In absence of microbial support, synthesis of nanoparticles requires intensive energy, costlier and produces toxic chemicals but nanoparticles synthesized through in microbial system are energy intensive, cost effective, biocompatible, and ecofriendly. The wide microbial range of fungal, bacterial, viral and algal strain’s carbohydrates, proteins, and enzymes acts as capping agents and surfactants and shows capability to synthesis metal oxide, sulfide, metallic and nonmetallic nanoparticles in which these nanoparticles act as effective adsorbent for the bioremediation of wastewater pollutants and treatment [49]. The recent field of nano bioremediation or green synthesis of nanoparticles supports the treatment of wastewater from the contaminated areas through biogenically produced nanoparticles or materials synthesized from microbiological sources of nano size. Nano bioremediation is an economic and ecological approach and it has an advantage over chemical based synthetic methods due to the larger surface area, and volume ration concept, low size of the material for the reactions. economic and ecological aspects. The studies report the consequences and hazardous impact of heavy metals and metalloids which could be treated through biogenic nanoparticles [50]. The HMs can be easily removed from the wastewater using NM adsorbents. They have large specific surface areas with enhanced active sites which helps in better adsorption of contaminants [51]. Among the various water contaminants, HMs are a major environmental concern and the most dangerous, as they are highly toxic even in dilute concentrations, non-biodegradable, and accumulate throughout the food chain, destroying aquatic life and posing serious threats to human health [52]. HMs usually enter the food chain through aquatic floor and fauna. Plants tend to absorb heavy metals dissolved in water, and those metals accumulate in their roots and then move to edible parts like fruits and vegetables [53]. The terrestrial region of the sedimentary is rich in different metallic and non-metallic elements. However, the higher concentration of HM such as Cr, Cd, Ag, Pb, Cu, Ni, and Zn in water bodies causes pollution and physical-chemical changes. A higher level of HMs in water bodies causes detrimental effects in aquatic and terrestrial organisms, such as mutation, genotoxicity, generation of free radicals, oxidative stress, deactivation of enzymes or cellular machinery, and cell necrosis [17]. In the advanced approaches, the removal of HMs on the large surface area can be detected, adsorbed, and immobilized as contaminants by NMs. Nanomaterial adsorbents have many unique properties, such as nano-size, large surface area, highly reactive, strong solution mobility, strong mechanical property, porosity, hydrophilicity, dispersibility, and hydrophobicity characteristics [54]. Various types of NMs, such as bio sorbents, graphene, activated carbon, and magnetic NPs, have been investigated as effective adsorbents to remove heavy metal ions from raw wastewater. At the nanoscale, NMs often exhibit some special properties, such as a surface effect, a small size effect, a quantum effect, and a macro-quantum tunnelling effect [55]. These properties contribute to its extraordinary capacity for adsorption and reactivity, which are favourable for the removal of heavy metal ions. Carbon nanotubes (CNTs) have been extensively investigated over the last few decades and are reported to exhibit many outstanding properties, including optical, electronic, vibrational, mechanical, and thermal properties. In another research copper, NPs were synthesized from *Escherichia* sp. SINT7 [51]. These NPs were copper resistant. The use of *Pseudoalteromonas* sp. CF10–13 in the preparation of NPs provides an eco-friendly method for biodegradation. NPs having biosorption activity effectively interact with the HMs and form chelating compounds for their removal from contaminated sites [17].

Overall, the use of microbes to synthesis NT/NPs from microorganisms is effective in the removal of lead from contaminated wastewater. There are many methods and several organisms that are known to remove specific metals from wastewater. Some of them are discussed below.

(a) Removal of Chromium

**Figure 4:**
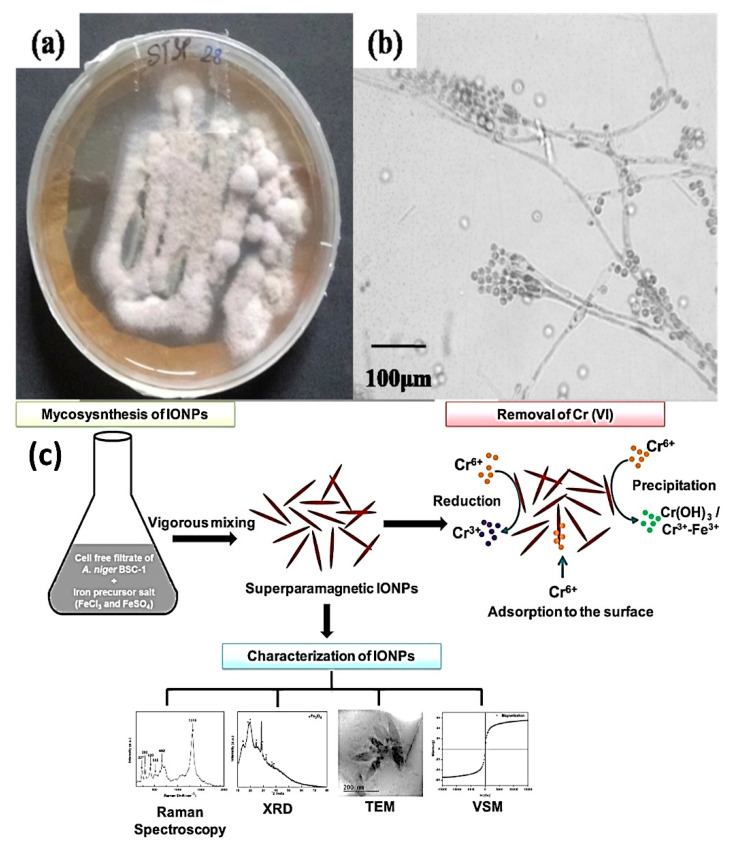
(**a**) This represents Manglicolous Fungal colony (**b**) Manglicolous fungi’s Compound microscopic image [60] (**c**) Desorption and reusability study, like efficiency of different regenerative solution for desorption of Cr, regeneration study. Reprinted/adapted with permission from Ref. [60]. 2022, Elsevier B.V.

The presence of chromium (Cr) in industrial effluents has become a major problem worldwide, as hexavalent chromium is highly toxic to animals due to its ability to generate reactive oxygen species in cells [56]. The trivalent state of chromium, on the other hand, is significantly less toxic and serves as an essential element in trace amounts. When industries such as electroplating, tannery, dyeing and others release their effluents into water bodies, hexavalent chromium enters the food chain and, consequently, reaches humans in a biomagnified form. In water bodies, Cr contamination and toxicity are the significant HM pollution challenge faced by researchers in terms of their mitigation [57]. Cr occurs most commonly in the trivalent/Cr(III) and hexavalent/Cr(VI) states. While the trivalent state of chromium is an important trace element, its hexavalent state is non-essential and toxic to animals and can cause dermatitis, lung cancer, kidney and gastric damage, and respiratory tract and eye irritation. Continued accumulation of toxic Cr(VI) along food chains often leads to biomagnification, putting humans at great risk. In one study, the biosynthesis of super-magnetic iron oxide NPs using the fungal strain *Aspergillus niger* BSC-1 has an extraordinary Cr(VI) removal efficiency [58]. This results in up to >99% selective removal of Cr(VI) ions by microfabricated NPs (Figure 4). Likewise, magnesium oxide NPs (MgO NPs) synthesized using *Aspergillus terreus* S1 were shown to significantly remove the Cr(VI) ion, i.e., 97.5% of wastewater. The conventional chemical reduction method comprises two steps—the reduction of Cr(VI) to Cr(III) by a reducing agent, at acidic pH, and the precipitation of Cr(III) as an insoluble hydroxide at alkaline pH. The chemical reductant can be any sulfur-based or iron-based salt [59]. These two steps can be combined into a single step by the electrochemical addition of a ferrous ion, rather than the addition of a ferrous salt (FeSO_4_ or FeCl_2_), which requires the use of an acidic and alkaline pH, making it a two-step process.

Adsorption is the most effective and economical solution for Cr(VI) remediation, especially if it is associated with adequate adsorbent regeneration. regeneration. In another system, the bio electrochemically active film of the Rhizobium-MAP7 strain helps in the detection and effective removal of Cr(VI) ions in the water system, is highly supported and recommended [61]. Another commonly used method involves the adsorption of Cr(VI) on various surfaces like titanium dioxide (Ti_2_O), goethite, activated carbon, zeolites and more. In some cases, reduction to Cr(III) may occur. Biosorption is a subdivision of adsorption, in which Cr(VI) is adsorbed on biomaterials found in abundance in nature, such as microbial biofilms. Adsorption is considered an effective method due to its low initial cost, flexibility in design and ease of operation. Furthermore, adsorption does not involve the formation of secondary residues such as sludge [62].

By implanting carbon nanofiber (CNF) anodes containing alumina (AA)/nickel (Ni) NPs in the double-chambered microbial energy component framework, the Cr(VI) has also been successfully removed, achieving power age *1540 mW/m^2^ [38]. However, for the hexavalent Cr, i.e., Cr (VI), the MFC system with the granulated activated carbon (GAC) filters with biogenic palladium NPs (Bio-PdNPs) significantly improved the Cr(VI)-removal and efficiency of the MFC [63]. In recent studies, *Shewanella oneidensis* was used for the Bio-PdNPs synthesis at the control parameter, these Bio-PdNPs with small dimensions act as a super catalyst and absorbent material for the complete reduction and removal of Cr(VI) from contaminated solution within a short period of 10 min up to five-time usage. Subsequently, the biogenic silicon NPs synthesized using the plant *Equisetum arvense*, i.e., green source to synthesize Si NPs (GS-SiNPs) as active adsorbent material for the significant removal of Cr (VI) from contaminated sites.

(b) Removal of Lead

An enormous increase in the application of various heavy metals including lead for commercial and non-commercial purposes has also led to their enhanced occurrence in the effluents from industries and domestic discharge creating substantial environmental concerns. Exposure of lead in the form of pipe corrosion, faucets, household plumbing systems, old paints, mining, smelting, battery manufacturing, and other industrial and urban waste causes lead to enter the water cycle [64]. Lead (Pb) is another toxic HMs predominantly present in industrial effluents and wastewater systems. Though it is present in a small amount in the earth’s crust, it is also released by anthropogenic activities such as mining, industrial operations, and fossil fuel burning. It has no biological role to play and it causes potential health risks among individuals, hence considered a priority metal of public health significance [52]. For Pb removal, sodium cations (Na^+^) are considered to have better exchangeability than HM. The pores present on the adsorbent surface help in making the significant area available for adsorption, and the diffusivity of the heavy metal ions based on pore size plays a controlling parameter undeciding the efficacy. The biosynthesised Palladium NPs (Pd NPs) of 10–20 nm dimension using algal strain *Spirulina plantensis* have been reported to possess 90% removal efficiency of Pb from contaminated sites under comprehensive physiological stress. However, the Iron oxide NPs (FeO_2_ NPs) bio-fabricated by using the fungus *Aspergillus tubingensis* demonstrated > 90% removal of not only Pb (II) but also other heavy metals, i.e., Ni (II), Cu (II), and Zn (II) from the contaminated sample. This approach also allows five times recyclability of the NPs used for remediation purposes.

(c) Removal of Mercury

Mercury (Hg) is also a HM with priority metal of public health significance mostly found in the aquatic system and effortlessly enters the food chain by sea food. Therefore, effective removal and remediation of the Hg from the wastewater have been done by the different approaches and different NPs at variable concentrations. For the removal of Hg, a hybrid catalytic membrane biofilm reactor (HCMBR) containing microflora such as *Azospirillum*, and *Delftia* were designed and evaluated as an effective system efficiently up to achieving 68.8 and 81.7%. In another study, the potential of biogenic selenium NPs (BioSeNPs) was investigated for the immobilization of Hg [25]. The study found that the salinity of the system along with the NPs aggregation resulted in reduced immobilization efficiency of Hg ion on the matrix. However, BioSeNPs coated with microbial biofilm on porous quartz resulted in enhanced retention and facilitated immobilization of Hg ions from the site contaminated with mercury selenide (HgS).

The effective and efficient removal of the toxic Hg(II), Ag-NPs embedded polyethylene glycol (PEG) nanocomposite functionalized by citrate was designed. This approach exhibited the potential for the accurate detection and removal of Hg^2+^, and excellent photocatalytic properties [38]. A cost-effective nanocomposite consisting of an Ag-Cu-dextran sensitized system with excellent photocatalytic properties can also be used for the selective and sensitive detection of Hg in wastewater. Biogenic Fe_3_O_4_ NPs of size 20 nm to 30 nm synthesized using the leaf extract of *Thymus schimperi* have exhibited significant removal of Hg (II) and can be reused without any significant loss of removal efficiency [65].

(d) Removal of Cadmium

Cadmium (Cd) is potent a metallohormone commonly poisonous at very low concentrations and a higher dose of Cd even after a short period of exposure results in severe toxic effects on health. The toxic effects of deliberate Cd exposure in the form of oxide, chloride, and sulfide also result in severe health issues due to cellular and molecular changes [66].

A wide range of NPs such as titanium dioxide (TiO_2_), Iron oxide (FeO), and Manganese oxide (MnO) have been studied for the effective removal of Cd from contaminated water bodies and provides significant outcomes [67]. The alumina NPs and glycerol-modified alumina NPs exhibited up to 99% of Cd (II) removal efficiency. A nanocomposite synthesized using peanut shell-derived biochar (BC) and the hydrated manganese oxide (HMO) has shown the potential outcome of 4–5 times the effective removal of Cd(II) as compared to the BC. Biogenic copper NPs (BioCuNPs) synthesized using a copper-resistant strain of *Shigella flexneri* SNT22 for an effective removal of Cd, it exhibited decreased acropetal Cd translocation by 49.62%. CuO NPs synthesized using agro-waste, i.e., orange peel and mint leaves have shown excellent metal adsorption (within 60 min) for Cd along with Pd and Ni [68].

**Figure 5:**
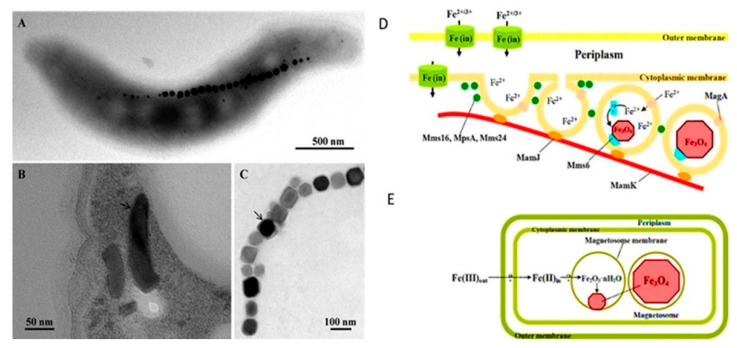
Transmission electron microscope (TEM) images of magnetosomes and the magnetosome membrane. (**A**) TEM micrograph of a cell of *Magnetospirillum magneticum* strain AMB-1 deposited onto a Formvar-coated electron microscope grid showing a chain of cuboctahedral magnetosomes. (**B**) TEM micrograph of an ultrathin section of a cell of “Ca. *Magnetoovum mohavensis*” showing the magnetosome membrane (arrow) surrounding bullet-shaped magnetite crystals. (**C**) TEM micrograph of an extracted and purified magnetosome chain from a *Magnetococcus marinus* MC-1 cell showing prismatic magnetite crystals surrounded by the magnetosome membrane (arrow). (**D**) Scheme of the hypothesized mechanism of magnetite biomineralization. (**E**) Model for magnetite biomineralization in *Magnetospirillum* species [70,71]. Reprinted/adapted with permission from Refs. [70,71]. 2022, Elsevier B.V.

Successful in situ removal of Cd has also been reported using biogenic iron oxide NPs (BioFeO-NPs) fabricated using an endophytic Bacterium *Pantoea ananatis*. The study also reported that this approach could prevent Cd bioavailability for the plant species MTB have potential application in the remediation of HMs as they can both passively align and actively swim along the magnetic field. Magnetosomes are MTB’s trademark nano-ranged intracellular structures, consisting of magnetic iron-bearing inorganic crystals encased by an organic membrane and serving as dedicated organelles for their magnetotactic lifestyle (Figure 5). MTB can absorb metal ions, including precious metal ions, hence their recovery and recycling is a sustainable way to decontaminate the wastewater from HMs [46]. MTB-based magnetic field technology has high application potential in wastewater treatment due to numerous advantages such as high efficiency, low power, low cost, and no secondary pollution, among others (Table 2). Among preliminary studies, researchers observed the application of MTB in environmental clean-up through metal loading. In these studies, up to 100% separation efficiency was achieved within 24 h. Further, Cd recovery by a sulfate-reducing MTB, *Desulfovibrio magneticus* RS-1, was investigated by and >95% of Cd at an initial concentration of 1.3 ppm was recovered during the operation [69]. Uncultured MTB from microcosms made gold and silver trapping possible. Thus, the research study showed that the adsorption of Au(III), Cu(II), and Ni(II) ions from aqueous solution by MTB in single and ternary systems had been investigated. These systems are eco-friendly, sustainable, and effective. They can be used on a large scale for the prospect.

### 2.2. Removal of Dyes

**Figure 6:**
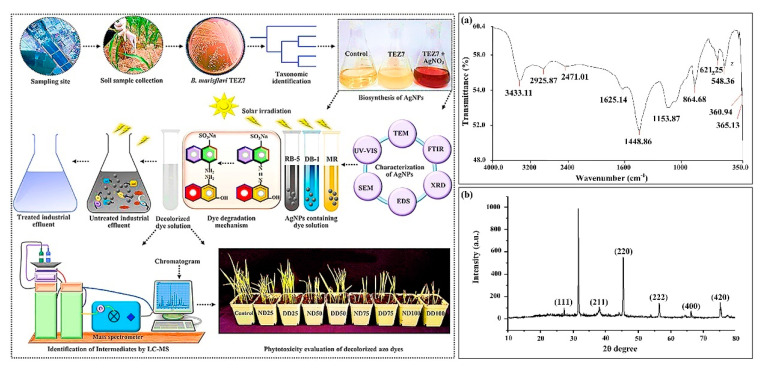
Silver nanoparticles transformed synthetic textile dye through LC-MS analysis and treated the actual wastewater. And Characterization of biogenic AgNPs by (**a**) FTIR spectra and (**b**) XRD analysis to measure the functional groups capping the NPs and to elucidate the crystal structure [78]. Reprinted/adapted with permission from Ref. [78]. 2022, Elsevier B.V.

The advanced oxidative wastewater pre-treatment methods yield several toxic by-products and expensive methodology of mineralization. Azo dyes are found to be very resistant to photo induced fading and biodegradation. The previous studies reported the combination of biodegradation and photocatalysis in which photocatalyst (g-C3N4-P25) and photosynthetic bacteria encapsulated in calcium alginate beads removed reactive brilliant red X-3b dye in waste water with efficiency of 94% and reduced the COD by 84.7% [77]. Many of the dyes discharged from the effluent of cosmetics, paper, paint, and tannery industries are non-degradable and hazardous waste for aquatic as well as terrestrial lives. Many important classes of dyes are aromatic halogenated compounds that are non-biodegradable, toxic, carcinogenic, and also mutagenic [38]. Various sustainable practices have been conducted in the past to remove dye instantly from aqueous solutions without any toxic byproducts. One of the approaches among many sustainable approaches is the use of biogenic Pd NPs synthesized by *Caldicellulosiruptor saccharolyticus* (a species of thermophilic, anaerobic cellulolytic bacterium) for the removal of dye (Methyl orange (MO) and diatrizoate) from water bodies that exhibited degradation efficiency significantly better than the chemically synthesized Pd NPs. The rate of degradation of dye by biogenic Pd NPs is twice that of chemically synthesized PdNPs. Besides, the released product of dye degradation is easily absorbed by the biogenic PdNPs to overcome and suppress the toxic effect [38]. PdNPs produced and dispersed by *Caldicellulosiruptor saccharolyticus* enhance the degradation of contaminants in water. In the same way, the catalytic efficiency of chemically synthesized and biogenic zinc oxide NPs (ZnO NPs) was compared for the removal of dyes such as methylene blue and reactive black-5 from contaminated water. ZnO NPs synthesized using *Pseudochrobactrum* sp. C5 indicated up to 95% removal efficiency for various dyes, i.e., methylene blue, brilliant blue R, reactive black 5, reactive red 120, and brilliant yellow, respectively significantly much better than chemically synthesized ZnO NPs 85.4%. Thus, the catalytic efficiency of biogenic ZnO NPs for the effective removal and remediation of dye from the aquatic system was much better than chemically synthesized NPs due to their diminutive particle size and larger surface area. Biogenic synthesis of silver NPs (AgNPs) for the transformation of a synthetic textile dye from the actual wastewater by functional groups capping the NPs and elucidating the crystal structure (Figure 6).

### 2.3. Removal of Pharmaceutical and Hospital Wastewater Pollutants

Recently, it was reported that the medical waste and pharmaceutical industry effluents contain many toxic xenobiotics and drug compounds that enter either directly into the aquatic system or through contaminated polluted sites [79]. Several conventional methodologies are used to assess a few common pollutants either qualitatively or quantitatively however, they do not apply to the estimation of complex toxic pollutants and their derivatives. The application of NT for the detection, analysis and remediation of such pollutants has become more convenient. Biogenic manganese oxide NPs (MnO NPs) synthesized using *Pseudomonas putida* to remove under in situ conditions indicate the maximum removal efficiency for steroids estrone and 7α-ethinylestradiol from the aqueous solution (Bharti et al., 2022). In biomedical waste, the drug molecule Diclofenac is an anti-inflammatory halogenated drug found in contaminated sites, and ozonation techniques are used to degrade these chemicals. However, only the partial removal of drug components occurs along with the release of a considerable number of noxious and mutagenic byproducts. In a study, the synthesis of Pd NPs by utilizing the metal-reducing bacterium *Shewanella oneiedensis* MR-1 has been helpful in the dehalogenation of aromatic compounds and acts as biogenic nanocatalysts [80]. However, in other studies, the bio-Pd NPs were doped with gold (Au) NPs to enhance their catalytic activity for removing diclofenac present in the hospital effluents wastewater treatment plants. In recent times the use of silver NPs (AgNPs) extracted from filamentous fungi (ascomycetes and imperfect fungi) and other fungal species has shown significant wastewater treatment properties. NPs effectively adhere to the cell membrane of microbial contaminants by damaging the cellular DNA [81]. They increase the cell permeability resulting in the generation of free radicles and reactive oxygen species. Besides, these nano-filters are very cheap to synthesize and have great efficiency in removing the pathogenic microbial load in the water. However, the removal rate of the microbial load was limited up to 4 min post-treatment of the samples. In another experiment a cement-silver nanocomposite concrete pebble was developed, that showed 99% removal of microbial load. Thus, the above-discussed approaches showed the importance of green NPs in the treatment of wastewater.

## 3. Bioaugmentation and Immobilization of Microbial Cells with Nanomaterials for Enhanced Wastewater Treatment

The debasement of unmanageable particles in the contaminated land and water bodies continues by utilizing a bioaugmentation microorganism. In-situ remediation of chlorinated ethenes in groundwater under anaerobic conditions by *Dehalococcoides* bacterium is a successful example of bioaugmentation in wastewater treatment. Besides that, other bacterial strains such as *Acinetobacter* sp. TW and *Comamonas testosteroni* I2 cause biodegradation of 4-fluoroaniline and 3-chloroaniline from polluted water [82,83]. Nevertheless, the highly effective outcome of bioaugmentation possesses certain limitations such as physiological parameters for the cultivation of microbial cells, and abiotic and biotic stresses (e.g., temperature, pH, lack of nutrients, phage infections, compatibility, and competitiveness) resulting in bioaugmentation failure.

To overcome the abovementioned limitation of immobilization of microbial cells, be a practical approach for stabilizing free-floating cell suspension and wastewater treatment. Immobilization technology is considered a potential solution to overcome the functioning of freely suspended microbial cells by providing a stable surface for adherence and reusability [84]. Various immobilization methods, viz. flocculation, cross-linking, encapsulation in a polymer gel, adsorption on NMs, covalent binding to nanocarriers, and entrapment in NM matrix, have been used for cell caging (Table 3). 

**Figure 7:**
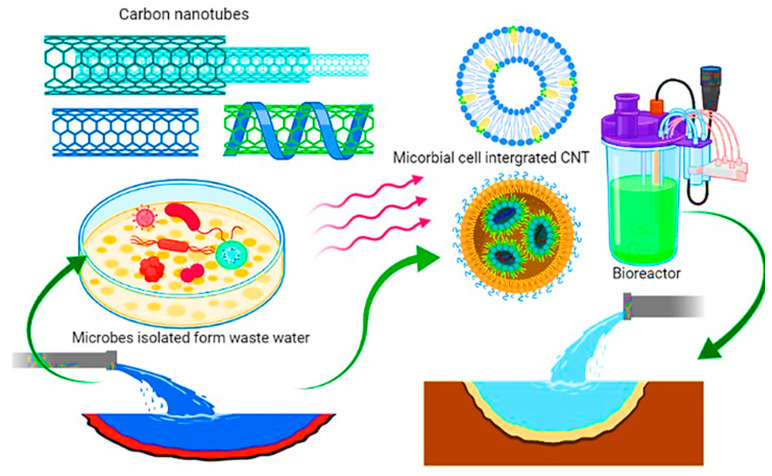
CNT-microbial integrated approach for waste water treatment and bioremediation of pollutants.

**Figure 8:**
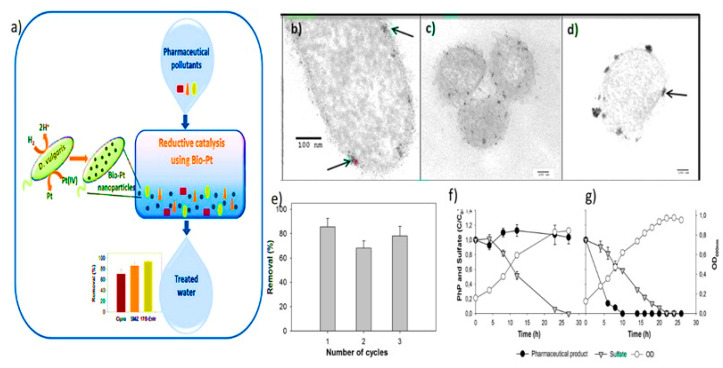
(**a**) This represents complete work flow removal of pharmaceutical compounds (**b**–**d**) TEM images of cell-supported palladium nanoparticles. (**e**) Removal of sulfamethoxazole by the Bio-Pt catalyst in three consecutive assays. Removal data obtained after 24 h of reaction. The error bars indicate the standard deviations of three independent experiments. The error bars indicate the standard deviations of three independent experiments. (**f**,**g**) Profiles of growth and removal of four pharmaceutical compounds (PhP) for 17β-estradiol and ibuprofen. The error bars indicate the standard deviations of three independent cultures [39]. Reprinted/adapted with permission from Ref. [39]. 2022, Elsevier B.V.

The high surface area to volume ratio of NMs provides better sorption of pollutants and biodegradation of pollutants by immobilized microbial cells. Therefore, many studies have focused on NMs for caging, and the immobilization of microorganisms for wastewater treatment indicated significant outcomes [91]. In a study, the calcium alginate-IONPs matrix used to immobilize *Phanerochaete chrysosporium* cells showed higher biosorption efficiency to remove Pb (II) from wastewater, i.e., 96.03% removal at pH five and 35OC. Subsequently, in other studies, NMs such as chitosan nanofibers, graphene nanosheets, silica NPs, and carbon nanotubes (CNTs) are utilized as matrices for microorganisms’ immobilizations and efficient removal of pollutants from contaminated water (Figure 7). The removal of pharmaceutical compounds by cell-supported palladium NPs and removal of sulfamethoxazole in the Bio-Pt catalyst in an assay for 24 h indicates the growth profiles removal of four pharmaceutical compounds (PhP) for 17β-estradiol and ibuprofen (Figure 8).

## 4. Integrated Microbial and Nanotechnological Approaches for Wastewater Treatment

### 4.1. Nanoparticle Integrated Microbial Reactor for the Removal of Pollutants from Wastewater 

Biogenic NPs synthesized by microbial cellular machinery is used in micro to macro reactor set-ups to enhance the removal efficiency of noxious and harmful contaminants from the treated effluents (Table 4). Many researchers also studied the stabilization of NPs on the bacterial cell walls and encapsulation technique on silica, polyacrylamide, polyurethane, and zeolites for reactor setups. Usually, the activated sludge used for the wastewater treatment results in effective outcomes for municipal wastewater but limited applicability for the remediation efficiency for industrial non-biodegradable and hazardous contaminants. Zero valent iron (ZVI) NPs are used to remove chlorinated organic compounds, and degradation of organic pollutants is an emerging aspect of wastewater treatment. ZVI NPs used in the bioreactor system with immobilized aerobic microbes to treat industrial wastewater have a double effect on removing pollutants. Nano bimetallic structures act as electron donors, causing partial digestion of the organic compounds to reduce them into non-toxic products by aerobic microorganisms.

The chlorinated solvents and petroleum hydrocarbons are degraded by granular iron by the dichlorination and oxidation into the chlorinated ethenes and short hydrocarbon chains. The synergistic effect of ZVI-NPs and bioactive components has been utilized to treat azo dye and nitroaromatic chemicals. A bench size experiment to investigate the effect of ZVI reactor followed by batch bioreactor for the biological treatment showed maximum levels of persistent organic products and heavy metals reduction. The BOD and COD level was 96.5% and 86% removal efficiency, along with nitrogen removal up to 52.2 from 35%, respectively. The study concluded that the ZVI reactor pre-treatment followed by the biological treatment compromises the most acceptable option for industrial wastewater purification.

### 4.2. Microbial Nanofibrous Webs Filters (NFW) for Wastewater Treatment

Another technology used for wastewater treatment is filtration and entrapment of pollutants through the membrane. However, extensive research has been conducted to design cost-effective and highly efficient filters for the treatment through nanotechnological aspects i.e., fabrication of electrospinning nanofibers/nano-webs. The NFW is suitable for the filtration and entrapment of micro to nano-dimension pollutants due to the nanoscale porosity and vast surface area [93]. Thus, wastewater purification and filtration can be enhanced by combining nanofibrous matrix with microbial cells such as bacteria or algae were studied and shown to have significant environmental implications owed significant impacts on environmental applications. In removing dye from wastewater, reusable electrospun CA-NFW immobilized bacterial strains *Clavibactermichiganensis*, *Pseudomonas aeruginosa*, and *Aeromonas eucrenophila* composite efficiently removed 95% of the dye molecules within 24 h.

In a study, a hybrid model of electrospun chitosan nanofiber mats (ECNMs) immobilized algal cells has been utilized to remove nitrates from wastewater. The ECNM-algal matrix is easier to handle, requires small space for cultivation, and shows up to 87% of nitrates removal from wastewater. However, the electrospun cellulose acetate nanofibrous webs (CA-NFW) immobilized ammonium oxidizing bacteria *Acinetobacter calcoaceticus* STB1 cells for ammonium removal from wastewater (98.5%) in the bacterial. Thus, NFWs are the less volume, more active surface area matrix for the immobilization of suspended free microbial cells and be a cost-effective reactor system for the remediation of toxic pollutants and heavy metals. Moreover, the biofilm of biogenic nanocomposite has high stability and significant activity under environmental stress.

### 4.3. Synergistic Combined Effect of Nanocomposites and Microbial Fuel Cells in Wastewater Treatment

In MFCs wide range of microbes are utilized as biocatalysts for the treatment of wastewater subsequently with the energy production from organic and inorganic pollutants. The bacterial cell helps in breaking down complex chemicals like acetate, propionate, and butyrate into simpler forms, i.e., H_2_O and CO_2_. Thereby the conventional technology for electricity production demand gets reduced by using the energy generated by MFCs. Although the components cost of MFC is much higher and performance is deprived than over all other types of fuel cells, it limits its applicability at the commercial level. Therefore, to reduce the treatment cost and utilization of cost-effective models such as membrane and cathode catalytic methods be the option for commercial-scale studies. The optimization of MFC activity in the material used for the reaction and digestion of substrate is a significant issue. There is a wide range of high catalytic activity materials and their modified forms used for the oxidative reduction reaction and to enhance the conductivity of the electrode surface [21]. NPs are the ultimate alternative material having specific interaction, and greater surface area interacts efficiently with the biological system for the entrapment and digestion of pollutants. Several nanocomposites and nanostructured carbon electrodes showed increased performance of MFCs. In a study, carbon nanotubes (CNT) with Pt electrodes act as a catalyst in MFC, and showed strong electrical conductivity in the COD media due to the electrode’s large surface area and higher catalytic efficiency. As a result, due to its unique features, the cathode catalyst used commercially for Pt in MFCs could be substituted by CNT/Pt. In another study, CNT nanocomposite with polymers such as polypyrrole (PPy) and polyaniline (PA) have been used to enhance microbial adhesion and reduce the NPs toxicity. The MFC anodes coating with CNT nanocomposites provides electrostatically bonded due to the presence of positively charged polycationic polymer. The active sites for microbial adhesion and electrochemical reactions are customized due to the PPy–CNT nanocomposite electrodes with a large surface area for bacterial attachment and biofilm formation for electron transfer to the anode surface. Thus, NM MFCs provides a better opportunity for electricity generation and treatment of wastewater.

### 4.4. Green Microbial Nanotechnology for Wastewater Treatment

**Figure 9:**
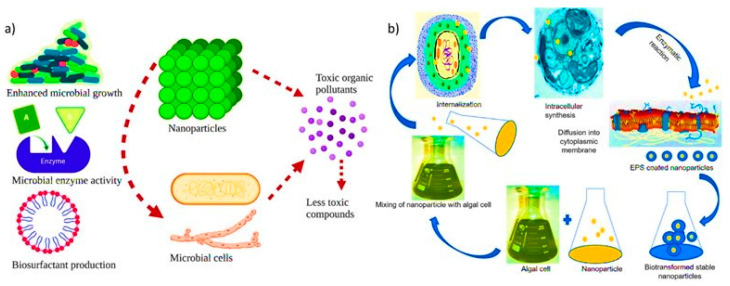
(**a**) Organic pollutant remediation using nanoparticles and microorganisms. Nanoparticles can be applied either directly or through adsorption to remove organic contaminants. Thereby facilitate bioremediation by enhancing the growth of microorganisms or by immobilizing contaminants through the production of microbial enzymes. (**b**) Exopolysaccharides-coated nanoparticle synthesis using algae [94]. Reprinted/adapted with permission from Ref. [94]. 2022, Elsevier B.V.

The bioremediation of wastewater through nanotechnological approaches, either by using NPs or combined treatment with microbial cells, results in efficient treatment processes and contaminating removal. The bio machinery, including bioactive micro molecules, nanosized catalytic membranes, nanotubes, nanopowder, nano-sorbents, and nanocatalysts, effectively entrap and remove the pollutants from wastewater, purify the system and improve water quality (Figure 9).

**Figure 10:**
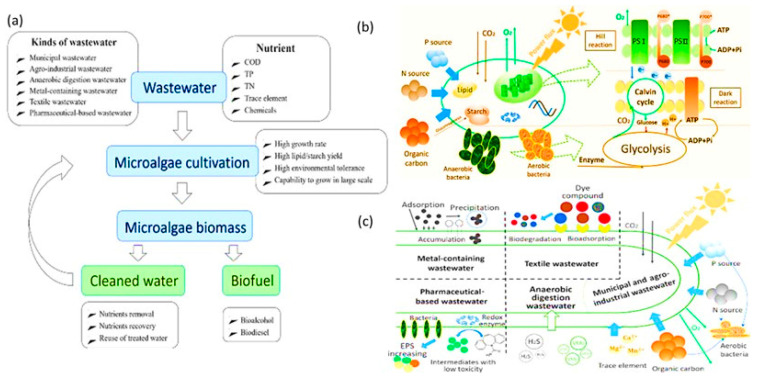
(**a**) Schematic presentation of simulations of wastewater treatment with microalgae biomass cultivation. (**b**) Nutrient and energy flow in microalgae-bacteria consortium. (**c**) Mechanisms of various wastewater treatments using microalgae and bacteria [101]. Reprinted/adapted with permission from Ref. [101]. 2022, Elsevier B.V.

**Figure 11:**
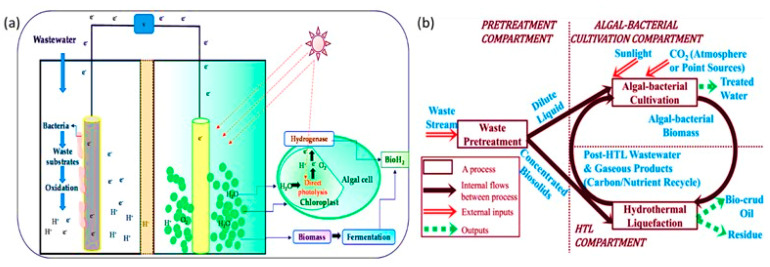
(**a**) Simplified schematic of the Environment-Enhancing Energy process for integrated wastewater treatment and biofuel production. (**b**) Microalgal-based microbial cells-based wastewater treatment and bioH2 production [102]. Reprinted/adapted with permission from Ref. [102]. 2022, Elsevier B.V.

In the green chemistry for wastewater treatment, algal membrane-based bioreactors with NPs were used as an integrated sustainable remediation and energy production technique. Here, the polluted wastewater bodies provide a nutrient-rich environment, i.e., micronutrients such as vitamins like thiamine and cyanocobalamin and macronutrients such as salts of PO_4_^3−^ and NO_3_^−^ with NH_4_^+^, Na, Ca, and K for the algal growth (Figure 10). Thereby the cultivation of algal cells in wastewater helps to remove the pollutants by adsorbing them [95,96]. There are several approaches for isolating algal biomass, either physically by centrifugation, air floatation, and sedimentation, or chemically by flocculation, nevertheless their higher cost for large-scale treatment. In the modern technology applicability of algal biomass for wastewater treatment and bioactive component production, the researchers have focused on developing integrated microalgae biorefineries (Figure 11). In this ingenuity, an algal-based algal membrane bioreactor (AMBR) for wastewater treatment with advanced modifications has been used to cultivate algal biomass at a high density and maximum absorption of contaminants [97]. In this approach, the maximum recovery of algal biomass with minimum harvesting parameters was obtained concerning the traditional methods. Synthetic polymers such as polysulfone (PSF), polyvinylidene fluoride (PVDF), and polyethersulfone (PES) are used for the fabrication of membranes that are chemically and physically stable [98]. However, a significant drawback in AMBR is membrane fouling, which will reduce the performance due to the deposition of particles or solutes in the membrane pores or the membrane surface. Usually, the hydrophobic membranes used to bind the hydrophobic group present on the microbial cell surface or solute particles causes membrane fouling. Another aspect is that the physicochemical properties of the membrane, such as surface charge, hydrophilicity, and roughness, can all affect the fouling of the reactor’s system [52,56]. The hydrophobic interactions between the pollutants and bioreactor membrane can be reduced by hydrophilic group generation to enhance the surface polarity by chemical or plasma surface coating and incorporating NPs. In a study, the reverse osmosis membrane outer layer of polyvinyl alcohol (PVA) was coated with TiO_2_, resulting in fouling reduction by introducing self-cleaning through UV irradiation [53]. Similarly, the hydrophilicity of the membrane surface was enhanced with antifouling properties and water flux by blending the PSF hollow fibre membranes (HFMs) with multi-walled carbon nanotubes (MWNTs) [84,85,86,87,88,89,90,91,92,93,94,95,96,97,98,99]. Metallic photocatalysis TiO_2_ has been investigated for various applications for pollution control systems and self-cleaning surfaces of the reactor system (Cruz et al., 2020). The hydrophilicity of the membrane gets enhanced by Nano-TiO_2_ due to the hydrophilic nature and high specific surface area for specific binding. Thereby membrane fouling reduces, and permeate flux increases. The TiO_2_-PVDF hollow fibre membrane with a single head-spinning inbuilt machine AMBR for wastewater treatment showed up to 75% of the free elemental phosphorus and nitrogen recovery, reduced membrane fouling due to the hydrophilic properties of PVDF in AMBRs [100]. Thus, incorporating NPs in AMBR demonstrated an effective model for wastewater recovery and overcoming membrane fouling without any chemical modification of the filter membrane.

## 5. Advancements and Future Approaches

With the discovery of advanced materials, including quantum dots, MXenes (metal carbides and nitrides), and borophene, the field of wastewater treatment has been revolutionized [103]. However, quantum dots possess a secondary threat due to possible leaching out into surroundings from treatment assemblies, which is hazardous to both humans and the environment. In contrast, advanced 2D materials such as MXenes, especially titanium carbide, possess excellent adsorption efficiency of around 90% to remove heavy metal ions and radionuclides from wastewater. Although such a remarkable efficiency, MXenes are yet to be adopted in commercial and practical practices due to several associated bottlenecks, including the use of corrosive acids during their fabrication, scalability, stability, and cost of production [20]. These bottlenecks can be easily addressed by adopting sustainable bio-nanotechnology-based routes such as microbial-based reduction of their precursors for fabrication, which will reduce the cost, complexity, and secondary contamination related to their manufacturing.

Furthermore, the plausible adoption of the microbial-based route for the synthesis of MXenes and similar emergent materials anticipates a sustainable, scalable, eco-friendly, and cost-effective approach for their fabrication, which opens new prospects for their wastewater treatment adoption [45]. Most importantly, it addresses the concern of leaching out of NMs into the environment as the green route synthesized NMs exhibit superior biocompatibility and negligible toxicity. Thus, one of the advanced prospects of green microbial bio-nanotechnology is to explore the fabrication of promising emergent NMs in wastewater remediation. Since the materials are in abundance, and the utilization of microbial-based fabrication strategies is significantly less, there is a necessity to explore more microbial-assisted synthesis routes for preventing secondary environmental contamination [51].

**Figure 12:**
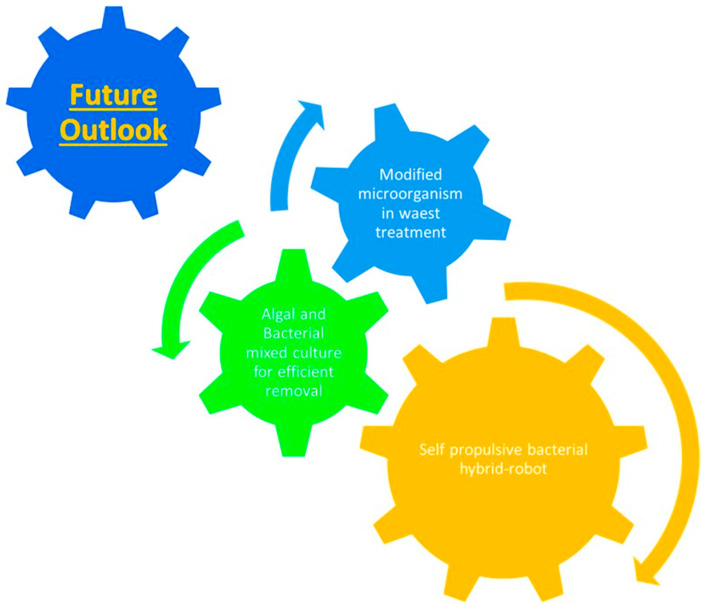
Future prospects of microbial based water treatment strategies.

Furthermore, the integration of advanced technologies, including internet-of-things (IoTs), artificial intelligence (AI), machine learning, cloud computing, and 5G communication with bio-nanotechnology-based water purification strategies, possess the potential to transform the conventional time-consuming and human resources consuming strategies [104]. The connectivity of wastewater treatment systems with advanced communication networks and IoTs, can be used for real-time monitoring and point-of-detection module. By training the systems using pre-analyzed algorithms and AI, the specific water contaminant can be detected, and a point-of-solution module can be devised employing a particular anti-contaminant NM. AI can be further used to explore the various microorganisms and their bio-reductants to fabricate various classes of NMs without undergoing actual experimental studies [105]. Hence, the integration of modern-day technologies with bio-nanotechnology and internet-of-nano-things possesses the potential to revolutionize the present-day water treatment strategies to meet global demand and sustainable development goals of clean water and hygiene (Figure 12). More research in this area is needed to isolate other MTB candidates and better understand the process of magnetosome formation and its role in pollutant removal from water bodies. If these challenges are overcome, MTBs will undoubtedly drive future nanotechnology advancements.

## 6. Conclusions and Outlook

This review explores various microbial nanotechnology-based strategies for wastewater remediation by removing a wide range of organic and inorganic contaminants. Microbial-assisted green NMs have exhibited remarkable absorption and removal efficacy towards recalcitrant and hazardous pollutants due to their high effective surface area, tunable physicochemical attributes, controllable variable morphology, superior regenerative capacities, and excellent performance biocompatibility, peculiar durability, and small intra-particular diffusion range. The utilization of microorganisms in the fabrication of NMs reduces the chemical load in the manufacturing process, and the biochemical involved act as reducing, capping and stabilizing agents. These microbial-assisted NMs exhibit superior adsorptive and catalytic efficiency than NMs fabricated using traditional chemical strategies. Although the notion of bioremediation by microbial NMs has been exhaustively evidenced, numerous formidable factors such as their scalable production, their stability for prolonged utilization, and lower exploration obstruct their commercial development for wastewater treatment. However, these issues have been addressed by adopting alternative strategies, including the immobilization of microbial cells on NM matrix and integrating NT with other green technologies such as algal-based membrane bioreactors, microbial fuel cells, aerobic digesters, and electrospun nanofiber webs. Exploring these alternative techniques is yet to be explored at a large scale with dedicated efforts to develop for commercial prospects.

Besides the commonly used microbes, MTB having nano-ranged intracellular structures has been studied for bio-mineralization. The application of immobilized cells and biogenic NPs in the bioreactor system provides an integrated system for the remediation and energy production from the contaminated effluents as a cost-effective approach with a significant outcome. Hence, based on this review and studies, in the future treatment strategies can be designed for the large sample bioremediation and clean-sustainable technology model designing.

Furthermore, additional studies are required to optimize bio-fabrication procedures concerning economic viability, culture, and handling of microorganisms. The integration of modern-day advanced technologies, including IoT, AI, 5G communication, and cloud computing with bio-nanotechnology-based green NMs is the near future of wastewater treatment strategies to meet the global concern of drinking water scarcity.

## Figures and Tables

**Table 2:** Different consortia of algal bacteria used in wastewater treatment.

Algal + Bacteria Strain		Type of Wastewater	Pollutant Removal			References
			COD (%)	Nitrogen (%)	Phosphorus (%)	
***Chlorella* sp.**	**+ activated sludge**	Synthetic wastewater	87	99	83	[72]
**Algal**	**+ bacteria**	Synthetic wastewater	96	46	45	[72]
***Chlorella vulgaris***	**+ activated sludge**	Municipal wastewater	55	95	100	[73]
***Scenedesmus* sp.**	**+ Bacteria group**	Municipal wastewater	92	95	98	[73]
***Selenastrum* sp.**	**+ bacteria**	Composting leachate liquids	NA	>90	92	[73]
***Chlorella vulgaris***	***+ Pseudomonas putida***	Synthetic wastewater	85	85	66	[74]
***Chlorella vulgaris***	**+ *Enterobacter asburiae***	Synthetic wastewater	95	100	96	[74]
	**+ *Klebsiella* sp.**	Synthetic wastewater	85	97	95	
	**+ *Raoultella ornithinolytica***	Synthetic wastewater	89	100	95	
	**+ *Pseudomonas putida***	Municipal wastewater	97	100	100	
	***+ Bacillus licheniformis***	Synthetic wastewater	86	88	80	
***Chlorella vulgaris***	**+ activated sludge**	Synthetic wastewater	83	89.4	91.4	[75]
**Consortium of algae, consisting primarily of *Chlorella*, *Chlamydomonas*, and *Stichococcus***	**+ bacteria**	Anaerobically digested swine manure	NA	NA	90	[76]
***Microcystis aeruginosa***	***+ Bacillus licheniformis***	Synthetic wastewater	65	21.56	70	[19]

**Table 3:** Substrates for the Immobilization of non-absorbent and effective removal of pollutants.

Pollutants	Matrix Composition	Microbial Strain	Removal Efficiency
Cu (II) Peng et al., 2010 [85]	Chitosan-magnetic NPs	*Saccharomyces cerevisiae*	96.8%
Diatrizoate [86]	PdNPs	*Shewanellaoneidensis* MR-1	77%
Nitrate [87]	Chitosan nanofibers	*Chlorella vulgaris*	87%
	Graphene nanosheets		9 mg L^−1^ day^−1^
Pb(II) [88]	Calcium alginate-IONPs	*Phanerochaetechrysosporium*	96.03%
Atrazine [88]	CNTs	*Arthrobacter* sp.	100%
Chlorophenol [89]	Magnetic NPs	*Rhodococcus rhodochrous*	90%
Decolourization of industrial effluents [90]	Silica NPs	Actinomycetes	80%

**Table 4:** Commercialized microbial NPs inbuilt bioreactor for wastewater treatment.

Bacterial Type	Nanoparticles	Microbial Strain	Pollutants	Removal Efficiency
Gram-negative facultative gamma proteobacterium [85]	PdNPs	*Shewanellaoneidensis* MR-1	Trichloroethylene	100% 98%
	Membranes integrating PdNPs	*Shewanellaoneidensis* MR-1	Diatrizoate	77%
	Bio-Pd beads bio-Pd polyurethane cubes	*Shewanellaoneidensis*	Trichloroethylene	98%
Gram-negative bacteria [85]	bio-Pd in MBR	*Citrobacter braakii* (ATCC 6750)	Diatrizoate	100%
Gram-negative, rod-shaped, saprotrophic soil bacterium [92]	MnONPs in MBR	*Pseudomonas putida*	Ibuprofen Naproxen Diuron Codeine N-acetylsulfamethoxazole Chlorophene Diclofenac Mecoprop	>95% >95% >94% >93% 92% >89% 86% 81%

## Data Availability

Not applicable.

## Abbreviations

AAS	Atomic Absorption Spectroscopy
AgNPs	Silver NPs
AMBR	Algal Membrane Bioreactor
CA-NFW	Cellulose Acetate Nanofibrous Webs
CNT	Carbon Nanotube
CrNPs	Chromium NPs
ECNM	Electrospun Chitosan Nanofiber Mats
EDS	Energy Dispersive Spectroscopy
EDX	Energy-dispersive X-ray spectroscopy
FTIR	Fourier-Transform Infrared Spectroscopy
HPLC	High-Performance Liquid Chromatography
MDNPs	Manganese Dioxide NPs
MFC	Microbial Fuel Cell
NT	Nanotechnology
NPs	Nanoparticals
NMs	Nanomaterials
PdNPs	Palladium NPs
IONPs	Iron Oxide NPs
PES	polyethersulfone
PSF	polysulfone
PVA	Polyvinyl Alcohol
PVDF	polyvinylidene fluoride
SEM	Scanning Electron Microscopy
TEM	Transmission Electron Microscopy
VSM	Vibrating Sample Magnetometer
XPS	X-ray Photoelectron Spectroscopy
XRD	X Ray Diffraction
ZnONPs	Zinc Oxide NPs
ZVI	Zero valent iron
